# DeMaSk: a deep mutational scanning substitution matrix and its use for variant impact prediction

**DOI:** 10.1093/bioinformatics/btaa1030

**Published:** 2020-12-16

**Authors:** Daniel Munro, Mona Singh

**Affiliations:** btaa1030-aff1 Lewis-Sigler Institute for Integrative Genomics, Princeton University, Princeton, NJ 08544, USA; btaa1030-aff2 Department of Computer Science, Princeton University, Princeton, NJ 08544, USA

## Abstract

**Motivation:**

Accurately predicting the quantitative impact of a substitution on a protein’s molecular function would be a great aid in understanding the effects of observed genetic variants across populations. While this remains a challenging task, new approaches can leverage data from the increasing numbers of comprehensive deep mutational scanning (DMS) studies that systematically mutate proteins and measure fitness.

**Results:**

We introduce DeMaSk, an intuitive and interpretable method based only upon DMS datasets and sequence homologs that predicts the impact of missense mutations within any protein. DeMaSk first infers a directional amino acid substitution matrix from DMS datasets and then fits a linear model that combines these substitution scores with measures of per-position evolutionary conservation and variant frequency across homologs. Despite its simplicity, DeMaSk has state-of-the-art performance in predicting the impact of amino acid substitutions, and can easily and rapidly be applied to any protein sequence.

**Availability and implementation:**

https://demask.princeton.edu generates fitness impact predictions and visualizations for any user-submitted protein sequence.

**Supplementary information:**

Supplementary data are available at *Bioinformatics* online.

## 1 Introduction

Amino acid variation across protein sequences, whether considering orthologous sequences across organisms or allelic variation across populations, is a fundamental aspect of protein evolution and function. Mutations within proteins can result in antibiotic resistance within bacteria (Woodford and Ellington, 2007), enhanced viral virulence (Geoghegan and Holmes, 2018) and inherited or acquired disease across individuals (Forbes *et al.*, 2011; Stenson et al., 2014). Thus, understanding the functional effects of amino acid substitutions represents a central challenge in molecular biology.

Experimental methods to characterize the effects of mutations within proteins have been performed for decades. Recently, deep mutational scanning (DMS) has enabled measurements of many or all possible single-residue substitutions within a protein in one experiment (Fowler and Fields, 2014). To date, this high-throughput approach has been applied to tens of proteins from a diverse range of organisms and has provided activity data for thousands of variants in each protein (Esposito *et al.*, 2019). While DMS is an exciting technology that continues to be applied to additional proteins, it is not currently feasible to use DMS to characterize all proteins of interest. This considerable knowledge gap necessitates the development of computational methods that can model and predict the impact of amino acid substitutions.

While numerous methods have been developed for variant impact prediction (e.g. see Hu *et al.*, 2019; Peterson *et al.*, 2013 and papers referenced therein), most have focused on predicting the effect of mutations on phenotypic outcomes at the whole-organism level, and have trained supervised machine learning models on collections of clinical mutation annotations in order to predict pathogenicity in humans (e.g. Adzhubei *et al.*, 2010; Carter *et al.*, 2009; Hecht *et al.*, 2015; Pejaver *et al.*, 2020; Rogers *et al.*, 2018). Alternatively, other methods have leveraged recent population-level sequencing data and have instead trained on human- and primate-derived alleles (e.g. Kircher *et al.*, 2014; Sundaram et al., 2018). Both of these types of approaches are geared toward pathogenicity predictions in human and are not applicable to protein sequences from other genomes. More recently, DMS data has been used in the context of supervised machine learning methods to predict the quantitative impact of amino acid substitutions on protein activity (Gray *et al.*, 2018; Klesmith and Hackel, 2019). In theory, these types of approaches are applicable to any protein sequence; however, in practice, most of these supervised machine learning methods—both pathogenicity and quantitative mutation impact predictors—utilize a wide assortment of protein features (e.g. conservation, predicted secondary structure and solvent accessibilty, etc.), and this limits their applicability to new sequences, as these features must be computed for new sequences and are instead often precomputed for genomes of interest. Alternatively, unsupervised methods for amino acid variant prediction rely only on aligments of homologous proteins and capture the effect of selection over long evolutionary distances (Choi *et al.*, 2012; Hopf *et al.*, 2017; Katsonis and Lichtarge, 2014; Kumar *et al.*, 2009; Riesselman *et al.*, 2018; Vaser et al., 2016). These methods include those that predict the impact of amino acid substitutions by considering just single sites at a time [e.g. SIFT (Kumar *et al.*, 2009; Vaser *et al.*, 2016) and PROVEAN (Choi *et al.*, 2012)], as well as those that model the interdependencies between positions within a protein [e.g. EVmutation (Hopf *et al.*, 2017), DeepSequence (Riesselman *et al.*, 2018) and GEMME (Laine *et al.*, 2019)]; the latter tend to be more effective than the former but are also more complex, harder to interpret and require larger numbers of homologous sequences.

Here, we introduce DeMaSk, a quantitative approach to predict the impact of amino acid substitutions, which combines the versatility of alignment-derived methods with empirically grounded protein fitness impact information as measured in DMS studies. Our main contributions are as follows. First, we use DMS data to infer an amino acid substitution matrix (AASM) that represents the average impact across sites of substituting one amino acid by another. We show that this AASM has notable asymmetries where the impact of changing from one amino acid to another is markedly different from the reverse; this asymmetry reflects the physicochemical properties of amino acids and is in contrast to AASMs commonly used for sequence alignments (Dayhoff *et al.*, 1978; Henikoff and Henikoff, 1992). Second, we train a linear model to predict the effect of an amino acid substitution at a site by combining the appropriate value from the DMS-derived AASM with two additional intuitive measurements: (i) the amino acid conservation across homologs at the site, which reflects the importance of the site to a protein’s function (Capra and Singh, 2007), and (ii) the frequency of the variant amino acid across homologs, which reflects whether the variant can be accommodated at the site in other proteins performing similar functions. Third, by testing on DMS datasets that are not used to fit the model, we demonstrate that while each of these three component measurements is by itself predictive of the impact of amino acid substitutions, the DeMaSk model combining all of them outperforms any of them individually. We also show that replacing the DMS-derived AASM feature within the DeMaSk model with a traditional, alignment-based AASM leads to worse performance. Fourth, we compare DeMaSk to several methods that use only homologous sequences to predict the impact of mutations, and find that DeMaSk performs as well as them; notably, we find that DeMaSk performs as well or better than cutting-edge, elegant but time-consuming methods based on undirected graphical models (Hopf *et al.*, 2017) and deep generative models (Riesselman *et al.*, 2018) that require numerous homologous protein sequences. We also demonstrate that our method DeMaSk considerably outperfoms a non-linear stochastic gradient boosting machine learning approach (Gray *et al.*, 2018) trained using DMS data along with numerous biological, structural and physicochemical features. Finally, we provide a webserver and open source software that takes as input any protein sequence and outputs predicted impacts for all possible amino acid substitutions, along with the individual contributions of the DMS-fit AASM, conservation values and variant frequencies to these predictions. Overall, we establish that DeMaSk is an effective, interpretable and easy to apply approach for predicting the quantative effect of missense mutations within protein sequences.

## 2 Materials and methods

*Overview*. DeMaSk uses data from mutagenesis experiments and homologous sequence alignments to model the impact of amino acid substitutions. In its first step, DeMaSk uses DMS variant fitness data to derive a directional AASM *D* where each entry $d_{i,j}$ represents the average impact on a protein’s functionality when substituting amino acid *i* with amino acid *j*. Second, for each sequence with DMS data, homologs are obtained and per-position conservation values and amino acid frequencies are computed. Third, a linear model is fit to the measured DMS data using as independent variables the per-position conservation values, the frequencies of the variant amino acids across homologs, and the appropriate values from the AASM. Finally, for a new query protein sequence, DeMaSk obtains its homologs, computes per-position conservation and variant frequency values, and uses these values along with the AASM and the fit coefficients to predict the functional impact of all possible substitutions in a query protein. DeMaSk does not rely on any information about a query protein aside from its sequence, requiring only that homologous sequences are available via database search.

###  

*DMS data used to compute substitution matrix and fit the linear model.* Publicly available data are collected from the studies listed in Supplementary Table S2 (Bloom, 2014; Brenan *et al.*, 2016; Doud and Bloom, 2016; Firnberg *et al.*, 2014; Giacomelli *et al.*, 2018; Haddox *et al.*, 2018; Heredia *et al.*, 2018; Kelsic *et al.*, 2016; Klesmith *et al.*, 2015; Mavor *et al.*, 2016; Melnikov *et al.*, 2014; Roscoe *et al.*, 2013; Roscoe and Bolon, 2014; Stiffler et al., 2015; Thyagarajan and Bloom, 2014; Weile et al., 2017; Wrenbeck et al., 2017). These include human, non-human eukaryote, bacteria and virus proteins, and all 380 amino acid substitutions are represented in each of these groups (Supplementary Table S3). Nonsense and synonymous mutations are removed. Within each selected dataset, the remaining per-variant values representing protein fitness are rank-normalized so that higher ranks represent higher fitness. The wild-type fitness quantile is then subtracted from all values, resulting in a uniform distribution of scores per dataset with a range of 1 and with 0 representing no fitness change. Usually, most of the values are negative while a smaller number of variants are gain-of-fitness and have positive values. Datasets are included only from DMS studies that measured all or nearly all possible amino acid substitutions in a protein so that rank-normalized scores have a consistent interpretation across proteins. Furthermore, DMS datasets are included only if the measure of fitness is related to the protein’s function [e.g. excluding studies that measured the effect of mutations on evasion of a host’s immune system (Ashenberg *et al.*, 2017)]. In cases where multiple datasets cover the same protein, the datasets are merged by averaging normalized fitness scores for the same variant. The normalized DMS value for a *wt* to *var* substitution in position *p* in sequence *s* is denoted by $\Delta \mathrm{fitnes}s_{s,p,wt,\mathrm{var}}$.

###  

*Computing the substitution matrix.* We use the normalized DMS data for each protein in Supplementary Table S2 to compute an asymmetric AASM *D* by averaging fitness scores for each of the 380 possible amino acid substitutions. That is, for amino acids *i* and *j*, we average the normalized values for mutation *j* at all positions where the wild-type amino acid is *i*. This results in values $d_{i,j}$ corresponding to the average impact of substituting amino acid *i* with amino acid *j*. Diagonal values are set to 0 since they represent no substitution. Because the measured DMS scores are rank-normalized per protein, the unit of fitness impact is the change in quantile across the fitness levels of all possible single-residue variants of a protein.

###  

*Obtaining homologs and computing per-position conservation scores.* DeMaSk identifies homologs for a protein sequence (whether the sequences with DMS data or a query sequence) using blastp with the UniRef90 database (The UniProt Consortium, 2015) and requiring an E-value threshold of 1e-5. Sequences are then filtered based upon their alignments to the query sequence so as to remove those that have <20% identity or >10% gaps in the alignments. After filtering, the top 500 BLAST hits are retained. The combination of blastp, UniRef90 and top 500 hits was chosen from among several choices of search algorithm, database and number of sequences considered because this combination has one of the highest performances on the training set and has reasonable runtime (Supplementary Fig. S1).

The residues aligned to each query sequence position are extracted from the blastp output and a multiple sequence alignment is built. For each position *p* in the alignment for sequence *s*, the number of times each amino acid occurs is counted and a pseudocount of $10^{-4}$ is added to these counts. Then, using these counts, the fraction $f_{s,p,aa}$ of times each amino acid *aa* occurs in each position *p* in the alignment built for sequence *s* is computed, with gaps excluded so that the 20 fractions sum to 1. To determine how conserved a site is, we compute the Shannon entropy of its distribution of amino acids (Shenkin *et al.*, 1991). Specifically, the Shannon entropy of the residues in position *p* in the alignment for sequence *s*, $H_{s,p}$, is computed as $-\sum_{aa\in \{A,C,\ldots ,Y\}}f_{s,p,aa}{\;\text{log}\;}_{2}f_{s,p,aa}$.

###  

*Model for predicting the impact of a mutation at a particular site.* For a sequence *s*, DeMaSk models the impact of a substitution at a position *p* as a linear combination of (i) the Shannon entropy of position *p* as computed across homologs of sequence *s*, (ii) the logarithm of the frequency with which the variant amino acid occurs across these homologs and (iii) the DMS-derived average impact for the particular combination of wild-type and variant residue identity, stored in the substitution matrix *D* computed above. That is, for a substitution from residue *wt* to residue *var* at position *p* in sequence *s*, we have

(1) $$\Delta \mathrm{fitnes}s_{s,p,wt,\mathrm{var}}=\beta_{0}+\beta_{1}H_{s,p}+\beta_{2}{\;\text{log}\;}_{2}f_{s,p,\mathrm{var}}+\beta_{3}d_{wt,\mathrm{var}}.$$

We use ordinary least squares regression to infer the coefficients using the 109 378 normalized and merged variant fitness scores from the datasets in Supplementary Table S2, along with the above substitution matrix and the homologous sequences found for each protein. Once fitted, the model can be applied to any variant in a query protein by finding homologs, computing the relevant position’s Shannon entropy and the frequency of the variant at that position, and combining those with the appropriate substitution matrix element. We note that it is possible to reformulate this regression so as to fit the elements of the AASM while also considering per-position Shannon entropy and variant frequency. However, in practice, we saw only small differences in performance between the two approaches, and chose the approach which computes the AASM elements directly, as this matrix is more interpretable.

###  

*Other methods to predict the impact of substitution.* We compare the performance of DeMaSk to three broad types of methods.

First, we compare DeMaSk to its three component features. To predict the impact of substituting residue *wt* with *var* in position *p* in the alignment for sequence *s*, the first method uses only conservation and returns $H_{s,p}$. The second method uses only the relative frequency of *var* at position *p*, and returns $log_{2}f_{s,p,\mathrm{var}}$. The third method returns the entry from the fitted directional AASM and returns $d_{wt,\mathrm{var}}$.

Second, we compare DeMaSk to PROVEAN (Choi *et al.*, 2012), EVmutation (Hopf *et al.*, 2017), DeepSequence (Riesselman *et al.*, 2018) and GEMME (Laine *et al.*, 2019), as these methods are, like DeMaSk, applicable to all protein sequences (e.g. not just human or model organism sequences), require as input only a single protein sequence or a multiple sequence alignment, can in theory report impact predictions for all possible substitutions within the input sequence, and are designed to predict the effect of variants on molecular function (e.g. as opposed to binary classification of deleteriousness); this is in contrast to most variant effect prediction methods (Hu *et al.*, 2019). We do not compare to SIFT (Kumar *et al.*, 2009), as it has been shown already to perform similarly to PROVEAN (Choi *et al.*, 2012). PROVEAN, EVmutation, DeepSequence and GEMME are unsupervised, and rely only on sequence homologs. More specifically, PROVEAN is a simple approach based on scoring the change in sequence similarity scores to homologs when introducing an amino acid mutation; its predictions of mutation impact in well aligned regions are based just on the site being mutated. EVmutation uses Markov random fields and explicitly incorporates parwise sequence dependencies across homologs. EVmutation also returns impact prediction scores under the assumption that all sites are independent of each other, and we refer to this version of the method as EVmut-Indep. DeepSequence considers higher-order dependencies via deep generative models. GEMME is a fast phylogentic method that considers interdependencies between all positions in a sequence. GEMME also returns impact prediction scores ignoring epistasis between sites, and we refer to this version of the method as GEMME-Indep.

Finally, we compare DeMaSk to Envision (Gray *et al.*, 2018), a stochastic gradient boosting machine learning method trained on DMS data and a diverse set of amino acid sequence features.

###  

*Evaluation and comparison to other methods.* In the primary analysis reported here, we test all methods on DMS datasets for proteins that are not used to derive DeMaSk’s directional amino acid substitution matrix (Supplementary Table S4) (Araya *et al.*, 2012; Bandaru *et al.*, 2017; Chan *et al.*, 2017; Diss and Lehner, 2018; Findlay *et al.*, 2018; Kitzman *et al.*, 2015; Matreyek *et al.*, 2018; McLaughlin *et al.*, 2012; Melamed *et al.*, 2013; Mishra *et al.*, 2016; Olson *et al.*, 2014; Qi *et al.*, 2014; Rockah-Shmuel *et al.*, 2015; Romero *et al.*, 2015; Sarkisyan *et al.*, 2016; Starita *et al.*, 2013; Wu et al., 2015; Zheng et al., 2018). We also perform leave-one-out cross-validation, where proteins in the training set are individually removed, the DeMaSk matrix and coefficients are fit from the remaining data, and performance is reported on the left out protein. For all testing, for each protein, we evaluate the predictions for each method by computing the Spearman’s rank correlation coefficient, or Spearman’s *ρ*, across measured variants between the predicted impacts and the DMS values as reported by the authors. We note that DeepSequence and EVmutation do not make predictions for all positions, and when comparisons are made to those methods, Spearman’s rank correlation coefficents for all methods are computed across only those positions for which predictions are made by DeepSequence and EVmutation.

###  

*Obtaining predictions for other published methods.* Each protein sequence was uploaded to the PROVEAN web server at http://provean.jcvi.org/seq_submit.php along with the list of all possible substitutions to be computed. PROVEAN was run with the default database, ‘NCBI nr, September 2012’. EVmutation predictions were obtained by uploading each protein sequence to https://evcouplings.org/job. The pipeline was run with default parameters, which includes parallel runs for alignment bitscore cutoffs of 0.2, 0.3 and 0.4. The EVmutation scores for the run resulting in the highest number of effective sequences were chosen for each protein. Since the EVmutation server accepted only query sequences up to 500 amino acids in length, we truncated longer sequences to the first 500 amino acids before submitting, with the exception of BRCA1 (residues 1–500 and 1364–1863 separately) Bgl3 (residues 2–501), Ube4b (residues 674–1173), PSD-95 (residues 102–601), PA (residues 1–500 and 217–716 to get predictions for residues 1–358 and 359–716, respectively) and MurJ (residues 1–500 and 12–511 to get predictions for residues 1–256 and 257–511, respectively). The alignments computed for each chosen run were downloaded and used as input to the DeepSequence standalone software (https://github.com/debbiemarkslab/DeepSequence). DeepSequence was run on GPUs using the default parameters provided for the examples included with the software. GEMME predictions were obtained by uploading protein sequence alignments to the web server at http://www.lcqb.upmc.fr/GEMME/submit.html using default options. The uploaded alignments were computed in the same way as for DeMaSk, but with a maximum of 5000 homologs each. For Envision, we downloaded precomputed predictions from https://envision.gs.washington.edu/shiny/envision_new/; this resource includes predictions for human and six model organisms, and covers 10 of the proteins in our test set, nine of which were not used in their training set.

## 3 Results

###  

**Fig. 1.**
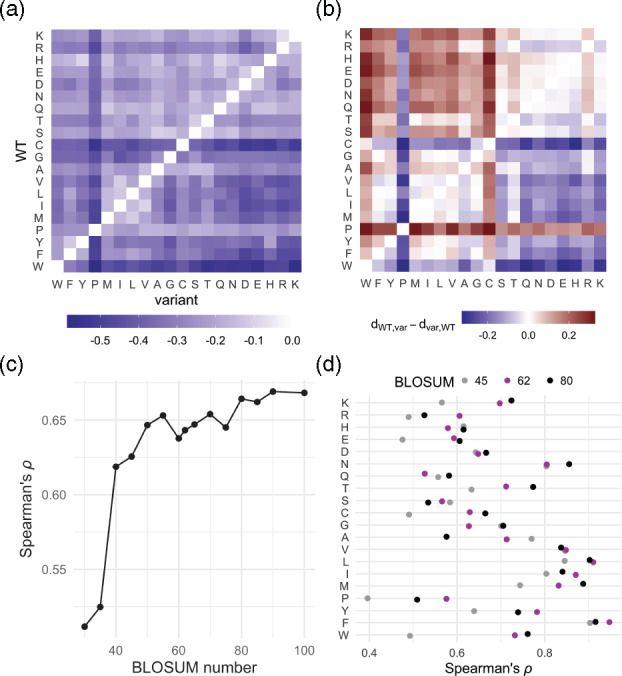
The DeMaSk mutational impact matrix. (**a**) The DeMaSk matrix fit from the DMS datasets in Supplementary Table S2. Entry *i*, *j* represents the average impact, across positions, on a protein’s functionality when amino acid *i* is substituted with amino acid *j*. Substitutions resulting, on average, in higher impacts are shown in darker colors. (**b**) To show the asymmetry per amino acid pair for the DeMaSk matrix, the matrix in (a) with its transpose subtracted from it element-wise is shown. Entry *i*, *j* is blue when the substitution from *i* to *j* is on average more detrimental than the substitution from *j* to *i*, and red when the substitution from *j* to *i* is on average more detrimental than the substitution from *i* to *j*. (**c**) Spearman’s rank correlation coefficient between the 400 elements of the DeMaSk matrix and the elements of each BLOSUM matrix. (**d**) The Spearman’s rank correlation coefficients between each row of the DeMaSk matrix and the corresponding row of BLOSUM45, BLOSUM62 and BLOSUM80

*Inferred directional substitution matrix has notable asymmetries.* We first fit the DeMaSk matrix, as described above, using the DMS data for 18 proteins (Supplementary Table S2), and analyze its features. Each entry *i*, *j* in the resulting matrix (Fig. 1a, Supplementary Table S1) corresponds to the average impact, across contexts (i.e. when not considering functional constraint as measured by homologous proteins), on a protein’s molecular function when substituting amino acid *i* with amino acid *j*. While the diagonal entries are fixed to be zero, other entries are negative and the DeMaSk matrix has clear asymmetries. For example, changes to Proline are more detrimental on average than changes from Proline; this is as expected since Proline imposes considerable constraint on the protein backbone and mutations to it would destroy local protein secondary structure (Morris *et al.*, 1992). Conversely, changes from Cysteine are more detrimental than changes to Cysteine; this is consistent with the fact that substitutions from Cysteine may disrupt disulfide bonds (Betz, 1993). To quantify the asymmetries in the DeMaSk matrix, for each *i*, *j*th entry, we subtract the *j*, *i*th entry (Fig. 1b). This reveals that changes from hydrophobic amino acids to polar and charged amino acids (bottom right of the matrix) tend to be more detrimental than the reverse (top left). Altogether, these asymmetries suggest that directional matrices are more appropriate for predicting the impact of amino acid substitutions than symmetric substitution matrices derived from alignments (Dayhoff *et al.*, 1978; Henikoff and Henikoff, 1992).

###  

*Notable differences between the directional DeMaSk substitution matrix and log-odds substitution matrices.* We next directly compare the DeMaSk matrix to the amino acid substitution matrices used to align protein sequences. Substitution matrices such as BLOSUM are derived from large sets of protein alignments and reflect the log-odds scores of the observed frequencies of pairs of amino acids substituting for each other, as compared to the expected frequencies (Altschul, 1991; Henikoff and Henikoff, 1992). The Spearman’s rank correlation coefficients between the DeMaSk matrix and BLOSUM substitution matrices varies, with higher correlations for the BLOSUM matrices numbered 50 or higher (Spearman’s rho = 0.638–0.670) than 45 or lower (Spearman’s rho = 0.512–0.626, Fig. 1c), as might be expected because higher-numbered BLOSUM matrices are computed from more similar sequences and are less influenced by long evolutionary distances. The correlations between the DeMaSk matrix and the BLOSUM matrices are all lower than those between any pair of BLOSUM matrices (Supplementary Fig. S2). We next consider correlations between the DeMaSk matrix and the BLOSUM matrices at the amino acid level. That is, for each amino acid, we compare the values for substitution to other amino acids between DeMaSk and the BLOSUM substitution matrices (Fig. 1d). For BLOSUM62, Spearman’s rank correlation coefficients vary from 0.526 to 0.947, with generally higher values for hydrophobic amino acids and somewhat lower values for some of the charged and polar residues (e.g. Glutamic Acid, Histidine, Glutamine and Serine). When comparing the correlations between the DeMaSk estimated matrix and the BLOSUM45, BLOSUM62 and BLOSUM80 matrices, we find that the BLOSUM45 matrix has the lowest Spearman’s rank correlation coefficients for more than half the amino acids. Further, just as in the whole matrix comparisons, in general, the DeMaSk estimated impacts correlate better for higher BLOSUM matrices than lower ones. Overall, our findings suggest that alignment-based substitution matrices contain biases introduced by long evolutionary timescales that are helpful for alignment-based applications but are not reflective of the immediate consequences of a given substitution.

###  

*DeMaSk predictions on DMS datasets as compared to component features.* We use the DMS-derived substitution matrix, along with per-position conservation values and the frequencies of the variant amino acids and train DeMaSk as described above. We test how well DeMaSk predicts the impact of substitutions by evaluating it on data from a set of deep mutational scanning experiments for proteins that are not included in the DMS data used to train DeMaSk. As a first step, we compare DeMaSk to its three individual components which predict variant fitness values as: (1) the corresponding matrix element from the DMS-derived directional AASM; (2) the conservation of the variant’s position as measure by Shannon entropy using the multiple sequence alignment of that protein and its homologs; and (3) the frequency of the variant in that position across the aligned homologs.

**Fig. 2.**
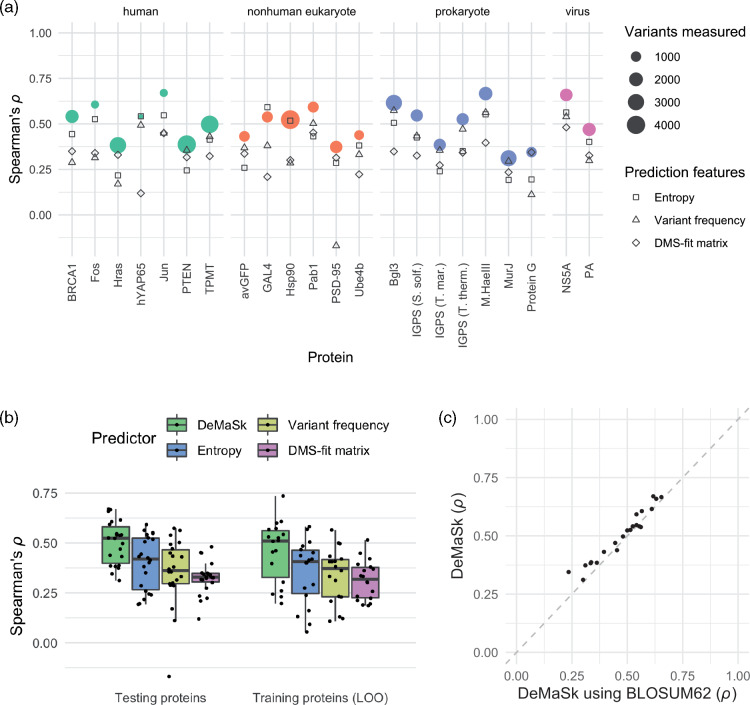
Performance on DMS fitness measurements. (**a**) Spearman’s rank correlation coefficients between measured fitness impact and prediction methods for test proteins grouped by organism of origin. DeMaSk performance shown in filled circles, with dot sizes indicating the number of single-residue variants measured in the original DMS study, and the performance of the three component features alone—conservation as measured by sequence entropy, variant frequency and the directional DMS-derived AASM—shown using squares, triangles and diamonds, respectively. (**b**) Boxplots of the per-protein Spearman’s rank correlation coefficients between measured DMS-measured fitness impact and predictions for test proteins (left) and training proteins using leave-one-out (LOO) cross-validation (right). (**c**) For each protein in the test set, the Spearman’s rank correlation coefficients between DeMaSk predictions and substitution impact scores is shown when using the typical DMS-fitted DeMaSk matrix (*y*-axis) versus when using the BLOSUM62 (*x*-axis) in its place within Equation 1. For nearly all proteins, higher performance is obtained using the DeMaSk matrix

The Spearman’s rank correlation coefficients of DeMaSk’s predictions on the set of 22 test proteins vary from 0.31 to 0.67 (Fig. 2a). For 20 proteins, DeMaSk’s predictions have higher Spearman’s rank correlation coefficients with the measured fitness values than any of its component features. For the remaining two proteins, conservation performs slightly better than DeMaSk. Across the proteins, DeMaSk’s predicted impacts have significantly higher correlations with the DMS data than those that arise when using only Shannon entropy, only the variant frequency, or only the directional AASM (Fig. 2b, left, *P*-values of 3.3e-6, 4.8e-7 and 4.8e-7, respectively, by the Wilcoxon signed-rank test). The median Spearman’s rank correlation coefficient for DeMaSk is 25.0% higher than the median correlation of conservation, which is the best of the component features. When considering performance on the set of 18 training proteins using leave-one-out cross-validation (Fig. 2b, right), we similarly find that DeMaSk outperforms its component features. Moreover, for all methods, the leave-one-out cross-validation performance is remarkably consistent with performance on the test set.

We next analyze the relative contribution of the three features to the DeMaSk linear model. We begin by noting that the learned *β* coefficients cannot be directly compared as the three feature values have very different ranges. Instead, for each experimentally measured substitution in the test set, we compute the contribution of each feature to the impact predicted by the DeMaSk model as the product of the feature value and the corresponding *β* coefficient in the linear model. We find that the contributions of the sequence entropy and DMS-fit matrix terms vary much more than the variant frequency term and their absolute values are larger as well (Supplementary Fig. S3). This suggests that the sequence entropy and DMS-fit matrix terms contribute more to differentiating amongst the predicted impacts, and even though the directional AASM feature correlates less strongly to observed fitness impacts than does variant frequency (Fig. 2b), it provides more unique information when all three features are used.

To test whether the DMS-derived AASM is effective for different categories of organisms, we group the training proteins into those from human, non-human eukaryotes, prokaryotes and viruses, and then compute the DMS matrix and train the DeMaSk model separately for each of these groups. We then group test proteins likewise and test each group using all of the trained models for each organism group. The organism-specific DMS-derived matrices have high correlation with each other and the learned models are no less capable of predicting on proteins of different organism groups than those of the same group, suggesting that DeMaSk effectively models protein properties that are shared even between organisms that diverged billions of years ago (Supplementary Fig. S4).

We then consider what happens to the performance of DeMaSk if BLOSUM62 is used in place of the DMS-based matrix when fitting our linear model (i.e. BLOSUM62 is used in place of $d_{wt,\mathrm{var}}$ in Equation 1). We find that for 20 of the 22 proteins, the DeMaSk model fit with the BLOSUM62 matrix results in lower correlations than when using the DMS-fit matrix (Fig. 2c). Using BLOSUM30 or BLOSUM90 instead of BLOSUM 62 results in even lower correlations across the proteins (Supplementary Fig. S5). Furthermore, adding BLOSUM62 (or BLOSUM30 or BLOSUM90) as a fourth feature to the DeMaSk model has minimal effect on performance (Supplementary Fig. S5). Altogether, these comparisons suggest that DeMaSk’s ability to predict variant impact comes from both sequence homologs and DMS data, and that matrices derived from DMS data are better suited than log-odds derived substitution matrices for this task.

###  

**Fig. 3.**
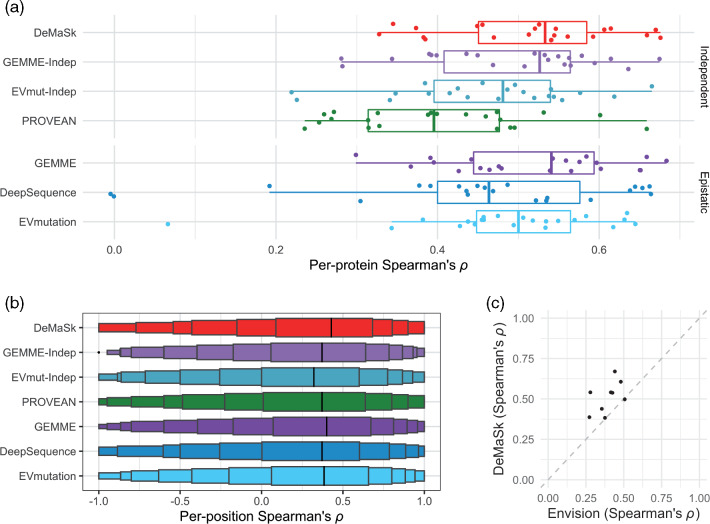
Comparison to existing prediction methods. (**a**) Boxplots of per-protein Spearman’s rank correlation coefficients between each method’s predictions and DMS-measured fitness scores. Methods are grouped by whether they use alignment positions independently (top) or consider epistasis (bottom). (**b**) Letter-value (or boxen) plots of per-position Spearman’s rank correlation coefficients between each method’s predictions and measured fitness scores. In letter-value plots, the widest boxes show half the data (from the 25th to 75th percentiles), while each successively narrower box shows half the remaining data. For (a) and (b), for all methods, only the positions for which EVmutation and DeepSequence provided predictions are used to compute correlations. (**c**) Scatter plot comparing the accuracy of DeMaSk to that of Envision. Correlations for Envision are computed only for the nine proteins in the test set for which precomputed scores were made available by the authors and which were not used to train the Envision model

*DeMaSk has state-of-the-art performance in predicting substitution impact.* We next compare DeMaSk to previous methods for predicting the impact of amino acid substitutions that use only alignment information. We first consider PROVEAN (Choi *et al.*, 2012), along with EVmut-Indep and GEMME-Indep, the versions of EVmutation (Hopf *et al.*, 2017) and GEMME (Laine *et al.*, 2019), respectively, that do not consider epistasis between sites; all three of these methods, like DeMaSk, consider each site independently. Across proteins, DeMaSk’s predictions tend to have higher Spearman’s rank correlations with the measured DMS values than the other three methods (Fig. 3a, top). Notably, DeMaSk considerably outperforms PROVEAN and EVmut-Indep.

We next compare DeMaSk to EVmutation (Hopf *et al.*, 2017), DeepSequence (Riesselman *et al.*, 2018) and GEMME (Laine *et al.*, 2019), methods that consider relationships between sites. Despite considering just a single site at a time, DeMaSk outperforms EVmutation and DeepSequence, which are sophisticated approaches based on Markov random fields and deep latent-variable models, respectively, and performs almost as well as GEMME, a phylogenetic approach requiring numerous homologous sequences for optimal performance (Fig. 3a, bottom). The Spearman’s rank correlations between predictions and DMS scores are given for all proteins and all methods in Supplementary Table S5, and show that the accuracies of all methods vary widely across the proteins.

DeepSequence’s predictions have correlations near zero on two proteins; while it has been noted previously that DeepSequence has relatively poor performance on viral proteins (Riesselman *et al.*, 2018), perhaps due to the lack of sequence diversity, these two proteins are bacterial and human (Supplementary Table S5). There are three proteins for which fewer than 500 homologs were retrieved by DeMaSk. While DeMaSk performs worse on these proteins as compared to its average performance on the other proteins, it nevertheless outperforms the other tested methods on each of the three (Supplementary Fig. S6). We note that PROVEAN, EVmutation, EVmut-Indep, DeepSequence, GEMMA and GEMMA-Indep use only protein sequence alignments, whereas DeMaSk also utilizes DMS data, which clearly gives it an advantage. On the other hand, DeMaSk is based on a simple linear model that is can easily be decomposed to extract the contributions of individual components, and performs similarily or better than these more complex methods.

We next consider how well the methods predict the impact of a mutation within each protein position; that is, for each position, we compute Spearman’s rank correlation coefficients between the DMS-measured impact of substitutions at that position with the predicted impacts. To avoid trivial correlations of -1 and 1, we only consider positions with at least three substitutions with measured and predicted impacts. We find that DeMaSk tends to better predict the ordering of variant fitness effects per position than all of the other methods (Fig. 3b), with Spearman’s rank correlation coefficients significantly higher for DeMaSk as compared to GEMME-Indep, EVmut-Indep, PROVEAN, GEMME, DeepSequence and EVmutation (*P*-values of 2.7e-18, 3.2e-37, 2.0e-26, 2.9e-5, 8.6e-22 and 4.5e-14, respectively, Wilcoxon signed-rank test). We note that the relative ordering of DeMaSk’s predicted impacts within a position do not depend on conservation, and thus are determined using only variant frequency across homologs and the AASM derived from DMS data on the training set.

Finally, we compare the performance of DeMaSk to Envision (Gray *et al.*, 2018), an earlier method that also leverages DMS data. DeMaSk has better performance for eight of the nine proteins in the test set for which Envision provides pre-computed impact scores (Fig. 3c, Supplementary Table S6). The superior performance of DeMaSk as compared to Envision is notable, as the latter is a gradient boosting machine learning method trained on DMS data and a diverse set of sequence features, whereas DeMaSk is based on a linear model that considers only conservation and amino acid substitutions.

###  

*DeMaSk web server and software.* DeMaSk predictions can be obtained for any user-provided protein sequence at https://demask.princeton.edu. The output file contains fitness impact predictions for every possible residue substitution, as well as the entropy, variant frequency, and DMS-fit matrix feature values used to compute each score. An interactive map of the predictions is shown on the results page to vizualize patterns across the protein sequence, and maps for each of the component features can be overlaid to compare their contributions to the predictions. DeMaSk software is also provided open source at https://github.com/Singh-Lab/DeMaSk. It computes predictions starting from a query sequence or from an existing multiple sequence alignment, and also allows the user to train the model on new data.

## 4 Discussion

We have developed an interpretable method, DeMaSk, to predict the impact of amino acid substitutions based on protein alignments and DMS data, generating a directional substitution matrix in the process. We have demonstrated that our method DeMaSk performs as well or better than substantially more complex methods for this task. Importantly, DeMaSk needs as input only a single protein sequence, and can provide predictions for every possible single residue substitution within it. Because DeMaSk treats alignment positions independently of each other, it can provide predictions even when relatively few homologs are found. This also allows for relatively short runtimes, with DeMaSk generating predictions for query sequences in the test set in 8 to 11.2 minutes each on a 1.6 GHz dual-core laptop. Almost all of this runtime is for finding homologs using blastp. Starting with aligned homologs, DeMaSk makes predictions on the test proteins in one or two seconds each.

Comparisons of DeMaSk to its component features reveals that sequence conservation by itself is a surprisingly powerful predictor of the impact of amino acid substitutions (Fig. 2), and indeed evolutionary conservation information has been found to be among the most useful features in more complex supervised prediction approaches (Gray *et al.*, 2018). The strong predictive ability of conservation suggests that the impact of different substitutions within a single protein position are highly correlated with each other. Thus, supervised machine learning methods, in their cross-validation testing, must not include substitutions from the same protein position in both training and testing sets, as this will lead to an overestimation of performance. More generally, we note that cross-validation testing of impact prediction approaches should put all variants for a single proteins in a fold together, as often neighboring amino acid positions are utilized to compute per-position features.

While DeMaSk and related methods clearly have predictive power in determining the effects of amino acid substitutions, they have important limitations as well. First, mutations may affect function via changes to a protein’s stability, interactions or other properties, and DeMaSk is not able to differentiate amongst these possibilities. Follow up computational structural studies may be helpful in shedding light on whether protein stability or interactions are affected. Second, DeMaSk shows a range of performance across proteins, and while factors such as alignment quality and the number of homologs used play a role in performance (Supplementary Fig. S6), it is not entirely clear when predictions are most accurate. While DeMaSk is fully automated, we believe that better performance may be possible in practice by curating alignments so that there is sufficient but not extreme variation across the homologs. Finally, while DeMaSk has state-of-the-art performance in predicting the impact of amino acid substitutions, for all proteins there is a clear performance gap when comparing DeMaSk’s predictions with experimentally measured impacts; new computational methods based upon DMS-derived data are likely necessary to achieve better performance.

A fruitful area for future work may be to combine rich probabilistic models of protein sequences (e.g. such as those that underlie EVmutation and DeepSequence) with DMS data. Indeed, these probabilistic models can be thought of as an elegant extension of methods that consider each amino acid position independently of the others, and measures that consider variation in the higher-order correlations between positions are likely to be beneficial within DeMaSk’s model. Alternatively, the directional AASM produced by DeMaSk may be useful as features within supervised machine learning methods to predict the impact of variants.

In the implementation of DeMaSk described here, a large amount of information from DMS experiments is condensed into one substitution matrix. Prior attempts to build such experimentally derived matrices (Yampolsky and Stoltzfus, 2005) were limited by the order of magnitude smaller number of measurements on the impact of amino acid substitutions. While our DMS-derived matrix is useful for gaining general insight into protein alterations and predicting substitution scores without requiring prior knowledge of the query proteins, this framework can also be utilized for more specific tasks. For example, if one wishes to obtain a transmembrane domain-specific substitution matrix for interpretation and prediction, the directional substitution matrix can be computed in the same way using DMS data filtered to transmembrane residues, provided sufficient data exist. Residue frequencies also vary according to depth within a protein’s core (i.e. distance from a protein’s surface), suggesting benefit from using an array of depth-specific directional substitution matrices (Farheen *et al.*, 2017; Yampolsky and Stoltzfus, 2005). Such specialized implementations will become more feasible as DMS datasets are generated for more proteins.

The directional substitution matrix derived from DMS data has potential uses beyond variant impact prediction. While purely alignment-based matrices such as BLOSUM are a logical choice for evolutionary investigations including detection of homology—as they reflect the frequency with which amino acids are observed in alignments—they are also often used to characterize functionally relevant chemical properties of proteins (Andreatta *et al.*, 2015; Gao *et al.*, 2010; Martínez-Jiménez *et al.*, 2017). We believe that such applications are better served by directional matrices, derived from experimental data, such as the one described here.

To conclude, we expect both DeMaSk and its underlying directional AASM to find wide usage. Toward this end, we have made DeMaSk available open source at https://github.com/Singh-Lab/DeMaSk and via a webform at https://demask.princeton.edu.

## Funding

This work was funded by the National Institute of Health (NIH) [R01-GM076275 to M.S. and T32 HG003284 to D.M.] and the National Science Foundation [DGE 1148900 to D.M.].

*Conflict of Interest*: none declared.

## Supplementary Material

btaa1030_Supplementary_Data
