# Supplementary material for: DeMaSk: a deep mutational scanning substitution matrix and its use for variant impact prediction
Source: Bioinformatics. 2020 Dec 16;36(22-23):5322–9. doi: 10.1093/bioinformatics/btaa1030 (PMC8016454; doi:10.1093/bioinformatics/btaa1030)
Supplement: btaa1030_Supplementary_Data [file btaa1030_supplementary_data.zip › supplement.pdf]

Supplementary Information for "DeMaSk: A deep mutational scanning substitution matrix and its use for variant impact prediction"

Supplementary Table 1

Supp_Table_1.tsv: The directional substitution matrix computed by DeMaSk.

Supplementary Table 2

Deep mutational scanning datasets used to compute the DeMaSk substitution matrix.

Protein	Organism	Study
NP	Influenza	Bloom 2014 [3]
MAPK1/ERK2	human	Brenan et al. 2016 [4]
H1	Influenza	Doud and Bloom 2016 [7]
TEM-1	E. coli	Firnberg et al. 2014 [9]
p53	human	Giacomelli et al. 2018 [10]
Env	HIV BF520, HIV BG505	Haddox et al. 2018 [11]
CCR5, CXCR4	human	Heredia et al. 2018 [12]
IF1	E. coli	Kelsic et al. 2016 [13]
LGK	Lipomyces starkeyi	Klesmith et al. 2015 [15]
ubiquitin	S. cerevisiae	Mavor et al. 2016 [17]
APH(3')II	E. coli	Melnikov et al. 2014 [20]
ubiquitin	S. cerevisiae	Roscoe et al. 2013 [27]
ubiquitin	S. cerevisiae	Roscoe and Bolon 2014 [26]
TEM-1	E. coli	Stiffler et al. 2015 [30]
H1	Influenza	Thyagarajan and Bloom 2014 [31]
CALM1, SUMO1, TPK1, UBE2I	human	Weile et al. 2017 [32]
amiE	P. aeruginosa	Wrenbeck et al. 2017 [33]

Supplementary Table 3

Supp_Table_3.tsv: Total counts for each wild-type/variant amino acid combination in the training data, as well as those counts when the dataset is split into human, non-human eukaryote, prokaryote, and virus proteins.

Supplementary Table 4

Deep mutational scanning datasets used to assess DeMaSk prediction accuracy.

Protein	Organism	Study
hYAP65	human	Araya et al. 2012 [1]
HRas	human	Bandaru et al. 2017 [2]
IGPS	S. solfataricus, T. maritima, T. thermophilus	Chan et al. 2017 [5]
Fos Jun	human	Diss and Lehner 2018 [6]
BRCA1	human	Findlay et al. 2018 [8]
GAL4	yeast	Kitzman et al. 2015 [14]
PTEN, TPMT	human	Matreyek et al. 2018 [16]
PSD-95	rat	McLaughlin Jr et al. 2012 [18]
Pab1	yeast	Melamed et al. 2013 [19]
Hsp90	yeast	Mishra et al. 2016 [21]
protein G	Streptococcus sp.	Olson et al. 2014 [22]
NS5A	Hepatitis C	Qi et al. 2014 [23]
M.HaeIII	H. aegyptius	Rockah-Shmuel et al. 2015 [24]
Bgl3	Streptomyces sp.	Romero et al. 2015 [25]
avGFP	Aequorea victoria	Sarkisyan et al. 2016 [28]
Ube4b	mouse	Starita et al. 2013 [29]
PA	Influenza	Wu et al. 2015 [34]
MurJ	E. coli	Zheng et al. 2018 [35]

Supplementary Table 5

Supp_Table_5.tsv: Spearman's rho between fitness scores measured in DMS studies and each method's predictions. The median and mean of rho values per method are listed first. The `rho_complete` values are for predictions on all DMS-tested variants in the protein, when available. The `rho_EV_DS_present` values are for the set of variants for which EVmutation and DeepSequence provided predictions. For proteins for which they provided all tested predictions, the value in `rho_complete` and `rho_EV_DS_present` are equal for each method.

Supplementary Table 6

Supp_Table_6.tsv: Spearman's ρ between fitness scores measured in DMS studies and DeMaSk's or Envision's predictions. The median and mean of the ρ values are listed first. Differences between DeMaSk's and Envision's median, mean, and ρ values are also given.

Supplementary Figure 1

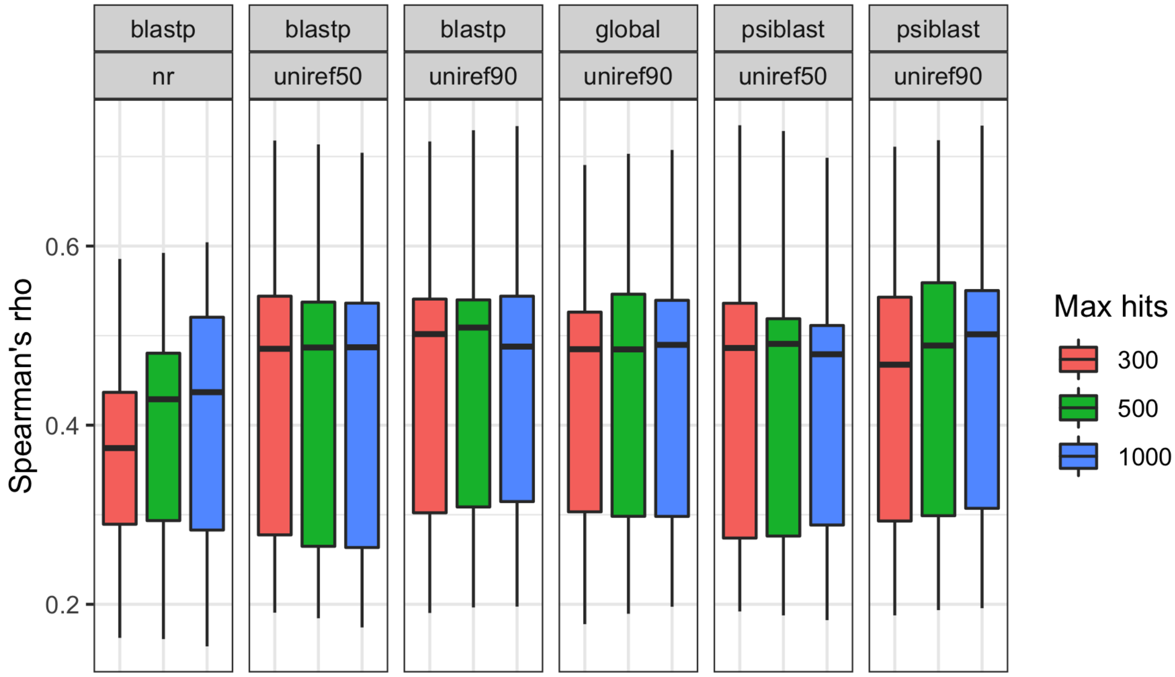

DeMaSk's performance, as shown by boxplots, when using different combinations of search and alignment algorithms (BLAST, PSIBLAST or BLAST followed by Clustal Omega), sequence databases (UniRef50, UniRef90, or the non redundant (nr) database), and maximum number of top hits used (300, 500, or 1000). DeMaSk model fitting and prediction are performed on training datasets using a leave-one-protein-out approach, since the testing datasets are subsequently used to report comparisons of the final DeMaSk model against existing prediction methods.

Supplementary Figure 2

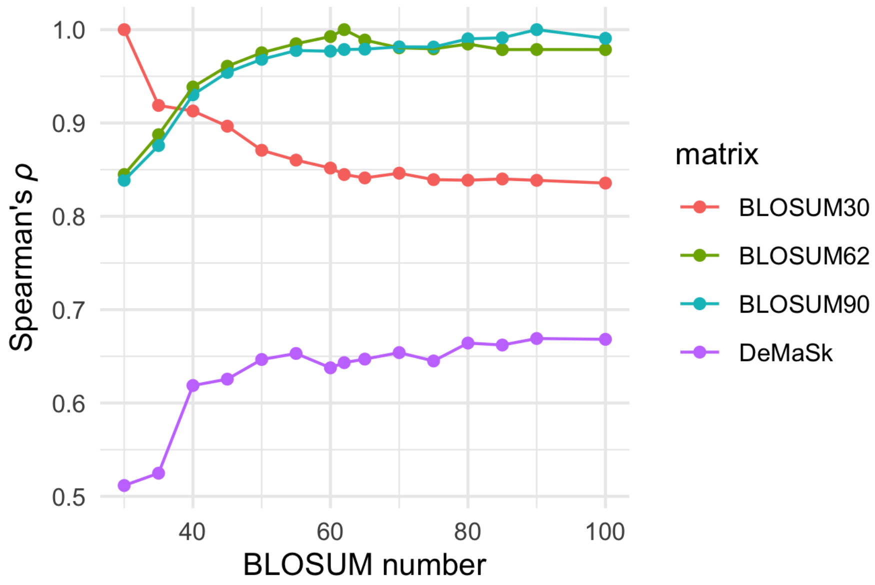

Spearman's rank correlation coefficient between the 400 elements of the DMS-derived matrix (purple) and the elements of each BLOSUM matrix (x-axis), as well as the correlations between BLOSUM30 (red), BLOSUM62 (green), and BLOSUM90 (teal) and every BLOSUM matrix for reference. This Figure is an extension of Figure 1c, and shows that, as expected, the DMS-derived matrix that DeMaSk uses is less similar to the BLOSUM matrices than are other BLOSUM matrices.

Supplementary Figure 3

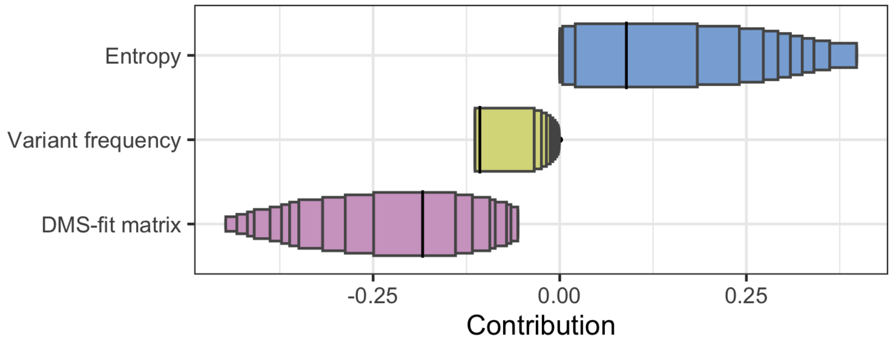

Letter-value (boxen) plots of the contributions of each DeMaSk feature (sequence entropy, variant frequency and DMS-derived matrix) towards the predictions of all measured substitutions in all test proteins. These are the terms of the linear model (i.e., the feature value times the corresponding linear coefficient from DeMaSk). The DeMaSk coefficients are 0.092 for Shannon entropy, 0.0051 for variant frequency, 0.76 for DMS-derived matrix, and -0.086 for the intercept.

Supplementary Figure 4

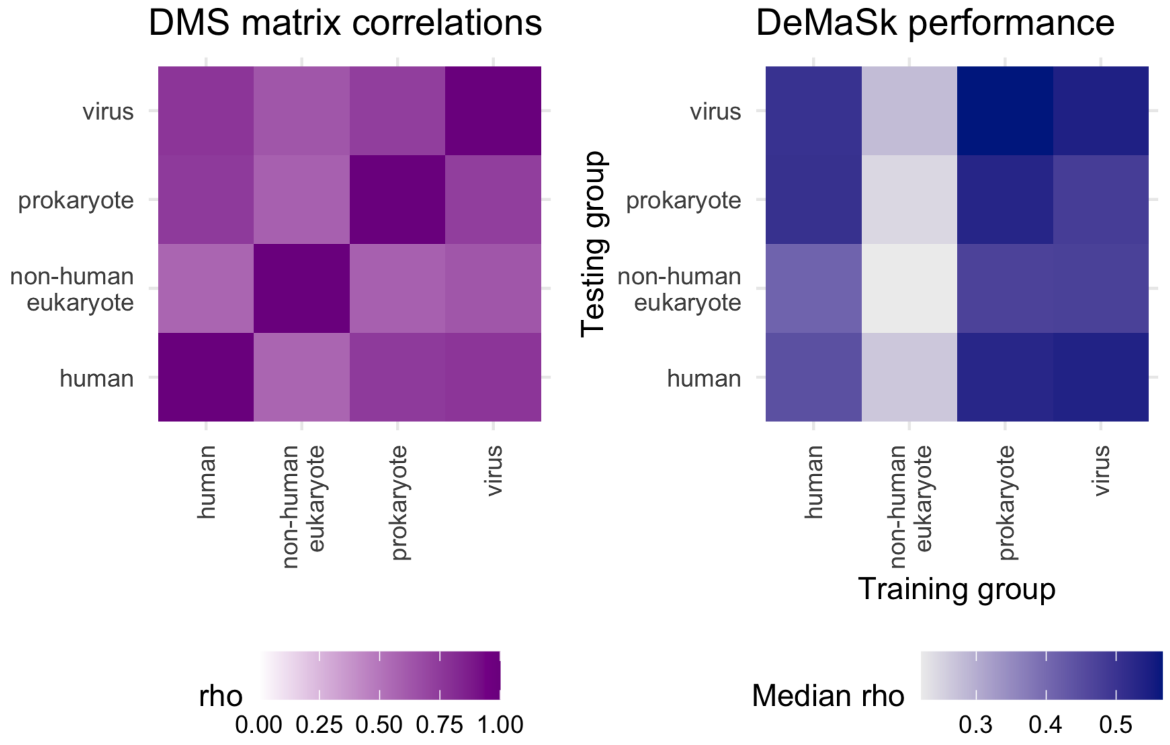

Results of splitting the training proteins into four organism groups and training the DeMaSk model separately on each group, and then testing each model on test proteins grouped in the same way. **Left:** Spearman correlation of the elements of each pair of group-specific DMS-derived matrices. **Right:** For each training (x-axis) and testing (y-axis) group pair, median Spearman correlation among the test proteins when DeMaSk is fit using data from the training group. Note that the non-human eukaryote training group contains only two unique proteins (four total datasets).

Supplementary Figure 5

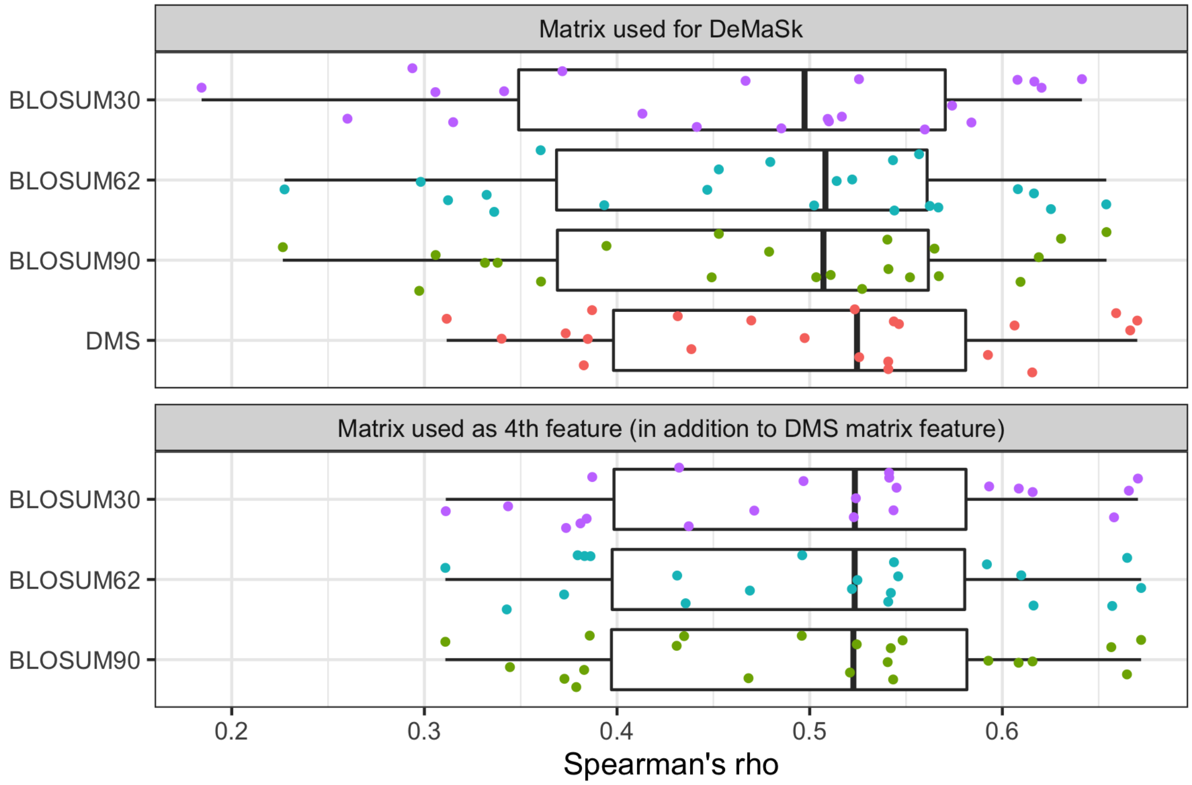

Performance on test datasets when using different substitution matrices for the DeMaSk model. **Top:** DeMaSk performance when using the DMS-derived matrix, BLOSUM30, BLOSUM62, or BLOSUM90 for the matrix feature. **Bottom:** DeMaSk performance when *adding* BLOSUM30, BLOSUM62, or BLOSUM90 as a fourth feature in the model.

Supplementary Figure 6

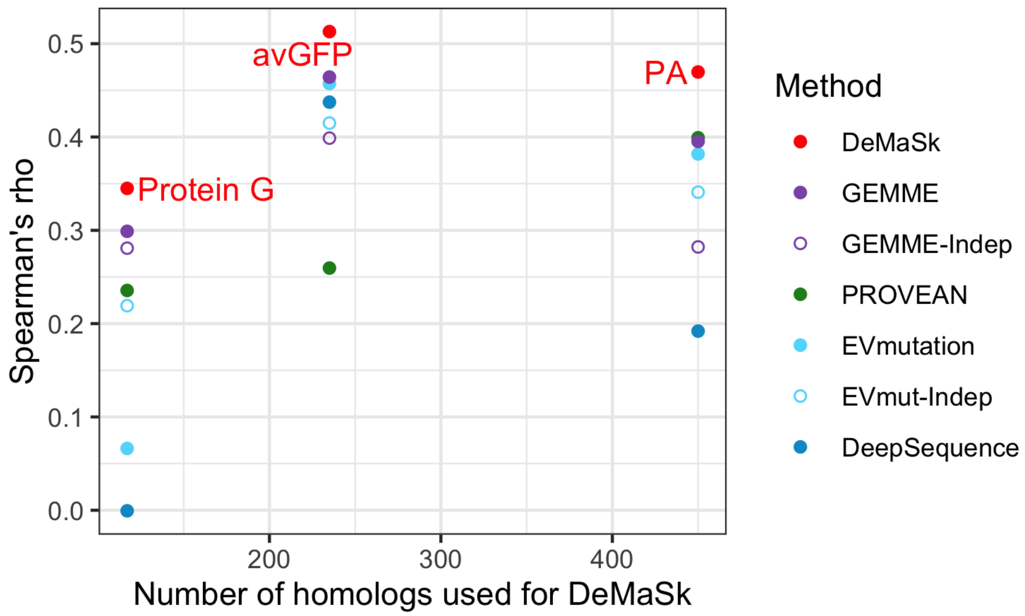

Performance of each prediction method on the three test proteins for which BLASTp finds fewer than 500 homologs in UniRef90. As in Figure 3, for all methods, only positions at which every method yields predictions are included.

References

[1] Carlos L. Araya, Douglas M. Fowler, Wentao Chen, Ike Muniez, Jeffery W. Kelly, and Stanley Fields. A fundamental protein property, thermodynamic stability, revealed solely from large-scale measurements of protein function. 109(42):16858–16863.

[2] Pradeep Bandaru, Neel H Shah, Moitrayee Bhattacharyya, John P Barton, Yasushi Kondo, Joshua C Cofsky, Christine L Gee, Arup K Chakraborty, Tanja Kortemme, Rama Ranganathan, and John Kuriyan. Deconstruction of the Ras switching cycle through saturation mutagenesis. 6:e27810.

[3] Jesse D. Bloom. An experimentally determined evolutionary model dramatically improves phylogenetic fit. 31(8):1956–1978.

[4] Lisa Brenan, Aleksandr Andreev, Ofir Cohen, Sasha Pantel, Atanas Kamburov, Davide Cacchiarelli, Nicole S. Persky, Cong Zhu, Mukta Bagul, Eva M. Goetz, Alex B. Burgin, Levi A. Garraway, Gad Getz, Tarjei S. Mikkelsen, Federica Piccioni, David E. Root, and Cory M. Johannessen. Phenotypic characterization of a comprehensive set of mapk1/erk2 missense mutants. 17(4):1171–1183.

[5] Yvonne H. Chan, Sergey V. Venev, Konstantin B. Zeldovich, and C. Robert Matthews. Correlation of fitness landscapes from three orthologous TIM barrels originates from sequence and structure constraints. 8:14614.

[6] Guillaume Diss and Ben Lehner. The genetic landscape of a physical interaction. 7:e32472.

[7] Michael B. Doud and Jesse D. Bloom. Accurate measurement of the effects of all amino-acid mutations on influenza hemagglutinin. 8(6):155.

[8] Gregory M. Findlay, Riza M. Daza, Beth Martin, Melissa D. Zhang, Anh P. Leith, Molly Gasperini, Joseph D. Janizek, Xingfan Huang, Lea M. Starita, and Jay Shendure. Accurate classification of BRCA1 variants with saturation genome editing. page 1.

[9] Elad Firnberg, Jason W. Labonte, Jeffrey J. Gray, and Marc Ostermeier. A comprehensive, high-resolution map of a gene's fitness landscape. 31(6):1581–1592.

[10] Andrew O. Giacomelli, Xiaoping Yang, Robert E. Lintner, James M. McFarland, Marc Duby, Jaegil Kim, Thomas P. Howard, David Y. Takeda, Seav Huong Ly, Eejung Kim, Hugh S. Gannon, Brian Hurhula, Ted Sharpe, Amy Goodale, Briana Fritchman, Scott Steelman, Francisca Vazquez, Aviad Tsherniak, Andrew J. Aguirre, John G. Doench, Federica Piccioni, Charles W. M. Roberts, Matthew Meyerson, Gad Getz, Cory M. Johannessen, David E. Root, and William C. Hahn. Mutational processes shape the landscape of TP53 mutations in human cancer. page 1.

[11] Hugh K Haddox, Adam S Dingens, Sarah K Hilton, Julie Overbaugh, and Jesse D Bloom. Mapping mutational effects along the evolutionary landscape of HIV envelope. 7:e34420.

[12] Jeremiah D. Heredia, Jihye Park, Riley J. Brubaker, Steven K. Szymanski, Kevin S. Gill, and Erik Procko. Mapping interaction sites on human chemokine receptors by deep mutational scanning. page ji1800343.

[13] Eric D. Kelsic, Hattie Chung, Niv Cohen, Jimin Park, Harris H. Wang, and Roy Kishony. RNA structural determinants of optimal codons revealed by MAGE-Seq. 3(6):563–571.e6.

[14] Jacob O. Kitzman, Lea M. Starita, Russell S. Lo, Stanley Fields, and Jay Shendure. Massively parallel single-amino-acid mutagenesis. 12(3):203–206.

[15] J. R. Klesmith, J. P. Bacik, R. Michalczyk, and T. A. Whitehead. Comprehensive sequence-flux mapping of a levoglucosan utilization pathway in E. coli. 4(11):1235–1243.

[16] Kenneth A. Matreyek, Lea M. Starita, Jason J. Stephany, Beth Martin, Melissa A. Chiasson, Vanessa E. Gray, Martin Kircher, Arineh Khechaduri, Jennifer N. Dines, Ronald J. Hause, Smita Bhatia, William E. Evans, Mary V. Relling, Wenjian Yang, Jay Shendure, and Douglas M. Fowler. Multiplex assessment of protein variant abundance by massively parallel sequencing. 50(6):874.

[17] David Mavor, Kyle Barlow, Samuel Thompson, Benjamin A. Barad, Alain R. Bonny, Clinton L. Cario, Garrett Gaskins, Zairan Liu, Laura Deming, Seth D. Axen, Elena Caceres, Weilin Chen, Adolfo Cuesta, Rachel E. Gate, Evan M. Green, Kaitlin R. Hulce,

Weiyue Ji, Lillian R. Kenner, Bruk Mensa, Leanna S. Morinishi, Steven M. Moss, Marco Mravic, Ryan K. Muir, Stefan Niekamp, Chimno I. Nnadi, Eugene Palovcak, Erin M. Poss, Tyler D. Ross, Eugenia C. Salcedo, Stephanie K. See, Meena Subramaniam, Allison W. Wong, Jennifer Li, Kurt S. Thorn, Shane Ó Conchúir, Benjamin P. Roscoe, Eric D. Chow, Joseph L. DeRisi, Tanja Kortemme, Daniel N. Bolon, and James S. Fraser. Determination of ubiquitin fitness landscapes under different chemical stresses in a classroom setting. 5:e15802.

[18] Richard N. McLaughlin Jr, Frank J. Poelwijk, Arjun Raman, Walraj S. Gosal, and Rama Ranganathan. The spatial architecture of protein function and adaptation. 491(7422):138–142.

[19] Daniel Melamed, David L. Young, Caitlin E. Gamble, Christina R. Miller, and Stanley Fields. Deep mutational scanning of an RRM domain of the Saccharomyces cerevisiae poly(A)-binding protein. 19(11):1537–1551.

[20] Alexandre Melnikov, Peter Rogov, Li Wang, Andreas Gnirke, and Tarjei S. Mikkelsen. Comprehensive mutational scanning of a kinase in vivo reveals substrate-dependent fitness landscapes. 42(14):e112–e112.

[21] Parul Mishra, Julia M. Flynn, Tyler N. Starr, and Daniel N. A. Bolon. Systematic mutant analyses elucidate general and client-specific aspects of Hsp90 function. 15(3):588–598.

[22] C. Anders Olson, Nicholas C. Wu, and Ren Sun. A comprehensive biophysical description of pairwise epistasis throughout an entire protein domain. 24(22):2643–2651.

[23] Hangfei Qi, C. Anders Olson, Nicholas C. Wu, Ruian Ke, Claude Loverdo, Virginia Chu, Shawna Truong, Roland Remenyi, Zugen Chen, Yushen Du, Sheng-Yao Su, Laith Q. Al-Mawsawi, Ting-Ting Wu, Shu-Hua Chen, Chung-Yen Lin, Weidong Zhong, James O. Lloyd-Smith, and Ren Sun. A quantitative high-resolution genetic profile rapidly identifies sequence determinants of Hepatitis C viral fitness and drug sensitivity. 10(4):e1004064.

[24] Liat Rockah-Shmuel, Ágnes Tóth-Petróczy, and Dan S. Tawfik. Systematic mapping of protein mutational space by prolonged drift reveals the deleterious effects of seemingly neutral mutations. 11(8):e1004421.

[25] Philip A. Romero, Tuan M. Tran, and Adam R. Abate. Dissecting enzyme function with microfluidic-based deep mutational scanning. 112(23):7159–7164.

[26] Benjamin P. Roscoe and Daniel N. A. Bolon. Systematic exploration of ubiquitin sequence, E1 activation efficiency, and experimental fitness in yeast. 426(15):2854–2870.

[27] Benjamin P. Roscoe, Kelly M. Thayer, Konstantin B. Zeldovich, David Fushman, and Daniel N. A. Bolon. Analyses of the effects of all ubiquitin point mutants on yeast growth rate. 425(8):1363–1377.

[28] Karen S. Sarkisyan, Dmitry A. Bolotin, Margarita V. Meer, Dinara R. Usmanova, Alexander S. Mishin, George V. Sharonov, Dmitry N. Ivankov, Nina G. Bozhanova, Mikhail S. Baranov, Onuralp Soylemez, Natalya S. Bogatyreva, Peter K. Vlasov, Evgeny S. Egorov, Maria D. Logacheva, Alexey S. Kondrashov, Dmitry M. Chudakov, Ekaterina V. Putintseva, Ilgar Z. Mamedov, Dan S. Tawfik, Konstantin A. Lukyanov, and Fyodor A. Kondrashov. Local fitness landscape of the green fluorescent protein. 533(7603):397–401.

[29] Lea M. Starita, Jonathan N. Pruneda, Russell S. Lo, Douglas M. Fowler, Helen J. Kim, Joseph B. Hiatt, Jay Shendure, Peter S. Brzovic, Stanley Fields, and Rachel E. Klevit. Activity-enhancing mutations in an E3 ubiquitin ligase identified by high-throughput mutagenesis. 110(14):E1263–E1272.

[30] Michael A. Stiffler, Doeke R. Hekstra, and Rama Ranganathan. Evolvability as a function of purifying selection in TEM-1 β-lactamase. 160(5):882–892.

[31] Bargavi Thyagarajan and Jesse D. Bloom. The inherent mutational tolerance and antigenic evolvability of influenza hemagglutinin. 3:e03300.

[32] Jochen Weile, Song Sun, Atina G. Cote, Jennifer Knapp, Marta Verby, Joseph C. Mellor, Yingzhou Wu, Carles Pons, Cassandra Wong, Natascha van Lieshout, Fan Yang, Murat Tasan, Guihong Tan, Shan Yang, Douglas M. Fowler, Robert Nussbaum, Jesse D. Bloom, Marc Vidal, David E. Hill, Patrick Aloy, and Frederick P. Roth. A framework for exhaustively mapping functional missense variants. 13(12):957.

[33] Emily E. Wrenbeck, Laura R. Azouz, and Timothy A. Whitehead. Single-mutation fitness landscapes for an enzyme on multiple substrates reveal specificity is globally encoded. 8:ncomms15695.

[34] Nicholas C. Wu, C. Anders Olson, Yushen Du, Shuai Le, Kevin Tran, Roland Remenyi, Danyang Gong, Laith Q. Al-Mawsawi, Hangfei Qi, Ting-Ting Wu, and Ren Sun. Functional constraint profiling of a viral protein reveals discordance of evolutionary conservation and functionality. 11(7):e1005310.

[35] Sanduo Zheng, Lok-To Sham, Frederick A. Rubino, Kelly P. Brock, William P. Robins, John J. Mekalanos, Debora S. Marks, Thomas G. Bernhardt, and Andrew C. Kruse. Structure and mutagenic analysis of the lipid II flippase MurJ from Escherichia coli. 115(26):6709–6714.
